# Successive Release of Tissue Inhibitors of Metalloproteinase-1 Through Graphene Oxide-Based Delivery System Can Promote Skin Regeneration

**DOI:** 10.1186/s11671-017-2305-4

**Published:** 2017-09-15

**Authors:** Cheng Zhong, Dike Shi, Yixiong Zheng, Peter J. Nelson, Qi Bao

**Affiliations:** 1 Department of Orthopaedics, First Affiliated Hospital, School of Medicine, Zhejiang University, Hangzhou, China; 2 Department of General Surgery, Second Affiliated Hospital, School of Medicine, Zhejiang University, Hangzhou, China; 3 Medizinische Klinik und Poliklinik IV, Campus Innenstadt, Clinics of University of Munich, Clinical Biochemistry Group, Schillerstr 42, 80336 Munich, Germany; 4 Department of Plastic and Reconstructive Surgery, Second Affiliated Hospital, School of Medicine, Zhejiang University, Hangzhou, China; 5 Institute of Gastroenterology, Zhejiang University, Hangzhou, China

**Keywords:** Graphene oxide, TIMP-1, Successive release, Skin regeneration

## Abstract

The purpose of this study was to testify the hypothesis that graphene oxide (GO) could act as an appropriate vehicle for the release of tissue inhibitors of metalloproteinase-1 (TIMP-1) protein in the context of skin repair. GO characteristics were observed by scanning electron microscopy, atomic force microscopy, and thermal gravimetric analysis. After TIMP-1 absorbing GO, the release profiles of various concentrations of TIMP-1 from GO were compared. GO biocompatibility with fibroblast viability was assessed by measuring cell cycle and apoptosis. In vivo wound healing assays were used to determine the effect of TIMP-1-GO on skin regeneration. The greatest intensity of GO was 1140 nm, and the most intensity volume was 10,674.1 nm (nanometer). TIMP-1 was shown to be continuously released for at least 40 days from GO. The proliferation and viability of rat fibroblasts cultured with TIMP-1-GO were not significantly different as compared with the cells grown in GO or TIMP-1 alone (*p* > 0.05). Skin defect of rats treated with TIMP-1 and TIMP-1-GO showed significant differences in histological and immunohistochemical scores (*p* < 0.05). GO can be controlled to release carrier materials. The combination of TIMP-1 and GO promoted the progression of skin tissue regeneration in skin defect.

## Background

Skin lesions can be caused by many factors such as accidents, diabetic complications, burns, or superficial surgery [1]. Autogenous skin transplantation, or biopolymers used for fabrication of the artificial skin, represents the most common approach used for wound closure [2]. These substitutes can include a limitation of available donor soft tissue, especially in severely burned patients [3], infection [4], pain, and skin flap necrosis [5], slowing healing and biocompatibility of the material.

Tissue inhibitor of metalloproteinase-1 (TIMP-1) prevents the extracellular matrix (ECM) from being decomposed by forming an inhibitory complex with the matrix metalloproteinases (MMPs) [6, 7]. TIMP proteins also control the MMP-driven turnover and processing of growth factors as well as cytokines linked to wound repair and regeneration [8]. TIMPs and MMPs are regulated during normal wound healing, and their imbalance has been implicated in skin repair defects, keloids, and fibrosis [9]. Epithelial-derived TIMP-1 can regulate epithelialization in different stages either directly or indirectly. An important phase of excisional wound repair and skin regeneration is re-epithelialization, which is the re-growth of epithelia over a traumatic surface [10]. Re-epithelialization occurs when cells at the wound margin loosen their cell–cell and cell–ECM contacts and begin to migrate across the wound. These processes have been linked to TIMP-1 biology [11].

An optimal regional TIMP-1 administration system for the restoration of the skin is dependent on the delivery vehicle used. Some delivery vehicles designed to release cytokines have been developed [12]. Carriers include PLGA (poly(lactic-co-glycolic acid)) [13], chitosan [14], PLGA nano-spheres [15], and hydrogels. The use of a delivery vehicle would help reduce the TIMP-1 dosage for skin regeneration and allow a regional delivery of the agent. Graphene is composed of carbon atoms with a flat monolayer and a honeycomb-like two-dimensional structure [16]. Graphene oxide (GO) has been used as a small molecule drug delivery vehicle in literature as it has efficient loading (absorption), is biocompatible, and has low toxicity [17–19]. Essential characteristics of GO include hydrophobic *π* domains in the core of the structure with ionized regions along the edges. The distinctive *π*–*π* stacking interaction makes GO efficient with water solubility, with a large specific surface area for high loading capacity [20, 21].

In the present study, we have investigated whether recombinant human TIMP-1 protein can be paired with GO as a delivery vehicle to improve skin regeneration. To investigate the effect on skin regeneration, TIMP-1 has been loaded onto GO flakes, and its release and toxicity are measured in vitro via rat fibroblasts. The results are finally tested on rats where a skin wound model is used.

## Materials and Methods

### Cell Culture

Rat fibroblasts were purchased from the Institute of Biochemistry and Cell Biology, CAS (Shanghai, China), and cultured in Dulbecco’s modified Eagle’s medium (DMEM, GibcoBRL, Gaithersburg, MD, USA) containing 10% (*v*/*v*) fetal bovine serum (FBS, Gibco). The medium was changed every 2 days. All the cells were kept at 37 °C.

### GO Characterization

GO flakes were purchased from Chengdu Organic Chemicals Co., Ltd. Chinese Academy of Science (Chengdu, China) and characterized using scanning electron microscopy (SEM, JSM-6701F, JEOL, Tokyo, Japan) after platinum coating. The samples were scanned under a scanning electron microscope (Hitachi S3000N) at 15 kV accelerating voltage. The size distribution of the GO flakes was determined with a zeta electric potential-based spectrophotometer (Zetasizer 3000 HSA, Malvern, UK). The morphology of the GO flakes was determined using atomic force microscopy (AFM, MultiMode, VEECO, USA) coupled with an inverted microscope (IX71 inverted microscope, Olympus, Tokyo, Japan). Thermal gravimetric analysis and differential scanning calorimetry were performed using a TGA/DSC thermogravimetric analyzer (Pyris 1 TGA, Perkin-Elmer, USA) by placing the samples in alumina pans and applying a heating ramp from 25 to 1100 °C at 10 °C/min.

### TIMP-1 Adsorption on GO

GO flakes were labelled with 1,1-dioctadecyl-3,3,3,3-tetram-ethylindocarbocyanine perchlorate (DiI, red, Sigma) before human recombinant TIMP-1 (Huaan Co., Hangzhou, China) adsorption. For TIMP-1 adsorption, fluorescein isothiocyanate (FITC, green, Thermo Scientific, Rockford, IL, USA)-conjugated TIMP-1 (Huaan Co., Hangzhou, China) and DiI-labeled GO were added to phosphate-buffered saline (PBS) and incubated for 4 h at 4 °C. The ratio of GO to recombinant TIMP-1 was 1:1 by weight. To determine the TIMP-1 loading on GO, TIMP-1 (1 μg) was added to 20 μl of PBS containing GO and incubated for 4 h at 4 °C. TIMP-1 adsorbed to GO was visualized using a laser scanning confocal microscope (IX81-FV1000 inverted microscope, Olympus). To confirm TIMP-1 adsorption onto GO, Fourier-transformed infrared spectroscopy (FTIR) was performed using a Nicolet 5700 spectroscopy (ThermoFisher Co., SGE, Australia) on pellets (10 mm in diameter) prepared by blending 2 mg GO with 100 mg KBr and pressing to produce the pellet to be analyzed. Spectra were analyzed after baseline correction by the software EZ OMNIC (Nicolet).

### Release Kinetics of TIMP-1 Protein

The release profiles of TIMP-1 from various GO concentrations (10, 20, and 30 μg/ml) were determined using a commercial human TIMP-1-specific enzyme-linked immunosorbent assays (ELISAs, R&D Systems Inc., Minneapolis, MN, USA). Following incubation for 4 h at 4 °C, an ELISA of the supernatant showed that virtually all the TIMP-1 was adsorbed on the GO. The TIMP-1-loaded GO was suspended in 60-mm culture dishes containing 1.5 ml PBS. The dishes were then incubated at 37 °C. At various time points, the supernatant was collected after continuous agitation and fresh buffer was added to the culture dishes. The concentration of human TIMP-1 in the supernatant was determined by ELISA.

### GO Biocompatibility Assay

Twenty micrograms per milliliter GO flakes loaded with a 20 μg/ml concentration of TIMP-1 were added to rat fibroblast cultures, and the cells were cultured for 6, 24, 48, and 72 h. Cell viability was evaluated by using the cell counting-8 (CCK8) assay as described in manufacturer’s instruction. The absorbance of the sample was expressed as absorbance value at 450 nm (*n* = 5 for each group).

### Flow Cytometric Characterization

Fibroblasts were harvested by trypsinization and labeled with Hoechst 33258 and Annexin-V-FITC/PI. Cell cycle activity and cell apoptosis were subsequently determined by flow cytometry analysis kit (Lianke, Hangzhou, China). Labeling was performed for 30 min at 4 °C in the presence of blocking reagent (Lianke, Hangzhou, China), followed by two washing steps using PBS. After washing and fixing, at least 10^4^ cells were acquired and analyzed. Flow cytometric analysis was performed using a Becton Dickinson FACSCanto II.

### In Vivo Experiment

All experimental procedures were conducted according to the guidelines of the NIH in the USA. The animals for experimental procedures were approved by the Zhejiang University Ethics Committee. Four-week-old male Sprague–Dawley rats were administered skin defect surgery (SDS) as described previously (Fig. 4a) [22]. The size of skin defection was 10 mm × 10 mm. After the surgery, the animals were returned to their individual cages. Fourteen days after SDS, the animals were randomly divided into four groups and therapy was initiated. Local injections of control agent (4 ml PBS only), GO agent (4 ml GO with PBS, 1:20), TIMP-1 agent (4 ml TIMP-1 with PBS 1:20), or TIMP-1-GO agent (4 ml GO with TIMP-1, 1:1 *v*/*w*) were administered subcutaneously. The injections were made every week around the skin defect (a total of 4 points, 1 ml each) for a total of two treatments over a span of 2 weeks. The rats were sacrificed 4 weeks after surgery (Fig. 4a). The regenerated skin was dissected, embedded in paraffin, and investigated by hematoxylin–eosin staining and Masson staining.

### Histological and Immunohistochemical Analysis

The regenerated skins were fixed in 4% formalin for 72 h. Then, the samples were decalcified in 9% formic acid for 2 weeks at room temperature. The samples of skin were dehydrated by graded ethanol. The consecutive sections were stained with hematoxylin–eosin (HE) and Masson. Expression of CD34 at the skin defect was analyzed by immunohistochemical staining. The sections were dewaxed in xylene and hydrated through graded alcohols. After blocking with 1% goat serum (1:100 dilution, Sigma), the sections were incubated with primary antibodies against CD34 (Abcam, Cambridge, UK) overnight at 4 °C. After washing with PBS for three times, the sections were incubated with secondary antibody for 1 h at 37 °C. Staining was developed with 3,3′-diaminobenzidine (DAB) solution (Dako, Hamburg, Germany). The regenerated skin was observed by three trained observers. The immunohistochemical sections were staged using the percentage of DBA.

### Statistical Analysis

All experiments were repeated three times, and the data were presented as means ± standard deviation. One-way ANOVA and the Student–Newman–Keuls post hoc test determined the level of significance. *p* values which are less than 0.05 and 0.01 were respectively considered to be significant and highly significant. Statistical analysis was performed with SPSS 17.0 (SPSS Inc., Chicago, USA).

## Results

### GO Characterization

**Fig. 1:**
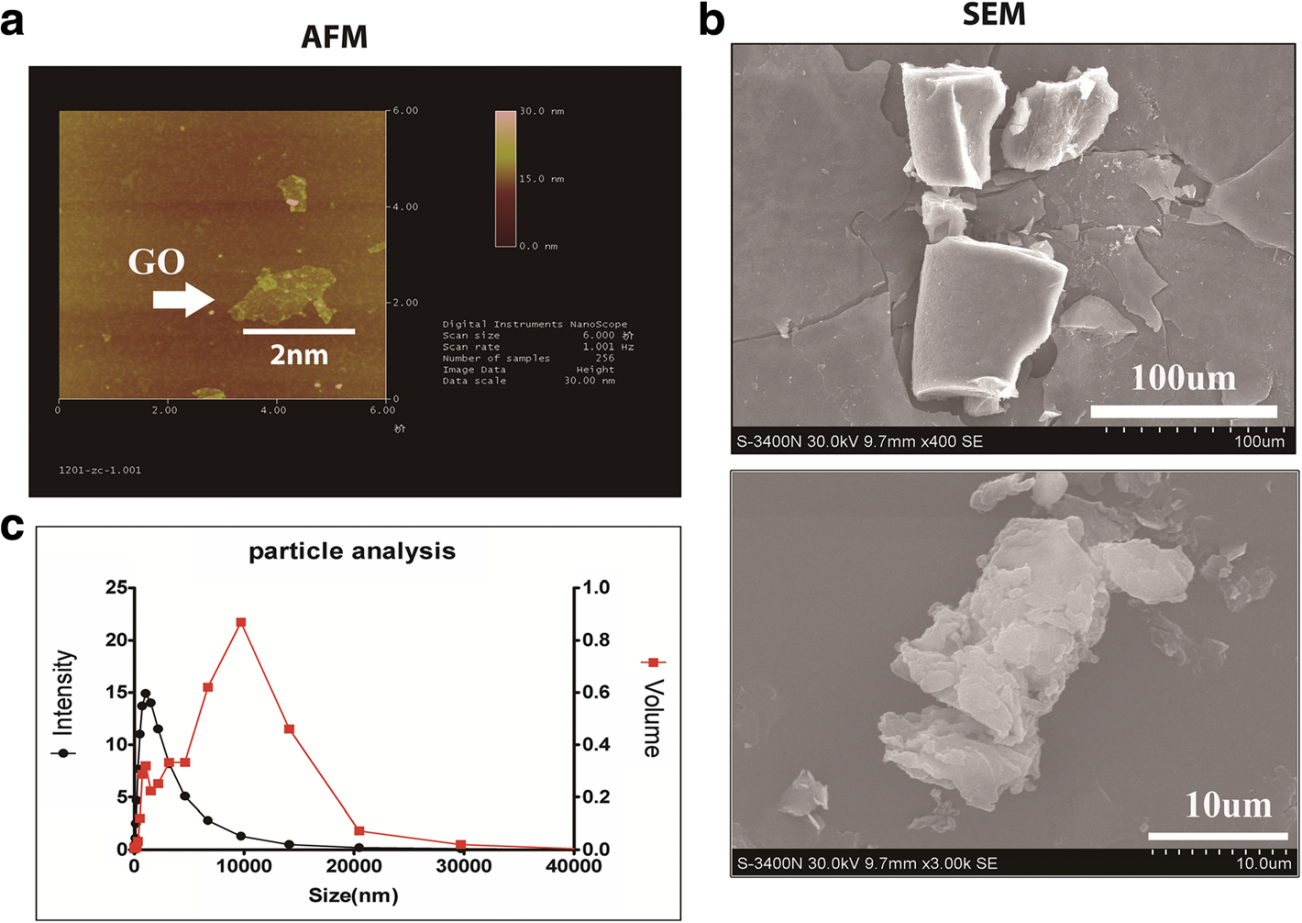
GO absorption. **a** 2D representation of GO images showed at AFM. **b** SEM shows that GO flakes are irregularly shaped sheets. The GO flakes were irregularly shaped sheets. **c** The size distribution of the GO flakes. The greatest intensity was 1140 nm and the most intensity volume was 10,674.1 nm

**Fig. 2:**
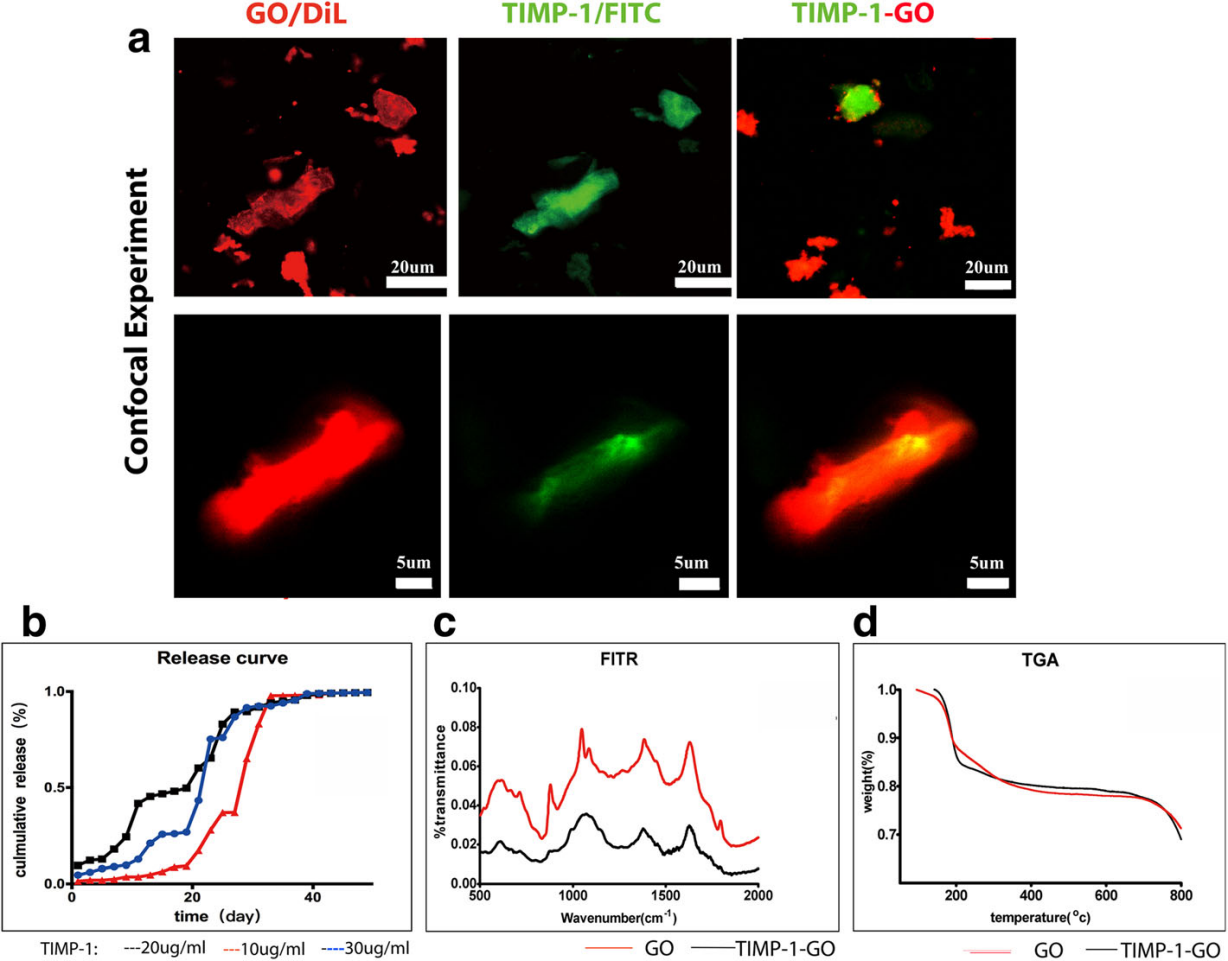
GO and TIMP-1-GO characterization. **a** TIMP-1 was absorbed onto GO. The analysis revealed that 75 ± 1.2% of GO was absorbed to TIMP-1. **b** The cumulative release profiles of TIMP-1 were recorded. TIMP-1 embedded in GO represents a suitable system for prolonged TIMP-1 release about 40 days. **c** The chemical composition between the GO and TIMP-1-GO was investigated using FTIR spectroscopy. The waveform and the wave peak of GO were significantly different from those of TIMP-1-GO. **d** The curve of thermal gravimetric analysis shows no major differences between GO and TIMP-1-GO from 50 to 800 °C. The curve of thermal gravimetric analysis showed no appreciable differences between control GO and TIMP-1-GO

The 2D representation of GO images is showed at AFM (Fig. 1a). A SEM showed that the GO flakes were irregularly shaped sheets (Fig. 1b). The size distribution of the GO flakes was measured by an electric potential-based spectrophotometer. The greatest intensity of size distribution was 1140 nm, and the most intensity volume of the GO was 10,674.1 nm (Fig. 2a).

### TIMP-1 Adsorption on GO

Following is the incubation of FITC-conjugated TIMP-1 (green) and DiI-labeled GO (red) in PBS for 4 h; TIMP-1 was adsorbed onto the GO. The analysis revealed that 75 ± 1.2% of GO was absorbed to TIMP-1 after 4 h, which suggested that GO efficiently binds to TIMP-1 protein (Fig. 1c). We investigated the chemical composition of TIMP-1-GO using FTIR spectroscopy (Fig. 2b). The waveform and the wave peak of GO were significantly different from those of TIMP-1-GO. We further investigated the thermal gravimetric analysis between control GO and TIMP-1-GO (Fig. 2d). The curve of thermal gravimetric analysis showed no appreciable differences between control GO and TIMP-1-GO.

### TIMP-1 Release

Various concentrations of TIMP-1 (group 1 3 μg/ml, group 2 2 μg/ml, and group 3 10μg/ml) were loaded onto GO. The cumulative release profiles of TIMP-1 are shown in Fig. 2c. The 2 μg/ml TIMP-1-GO release reached 50% cumulative release more rapidly as compared to the 10 and 30 μg/ml TIMP-1 dose. TIMP-1 was continuously released for about 40 days. This suggests that the application of TIMP-1 embedded in GO may represent a suitable system for prolonged TIMP-1 release (Fig. 2c).

### Cell Proliferation and Viability on TIMP-1-GO

**Fig. 3:**
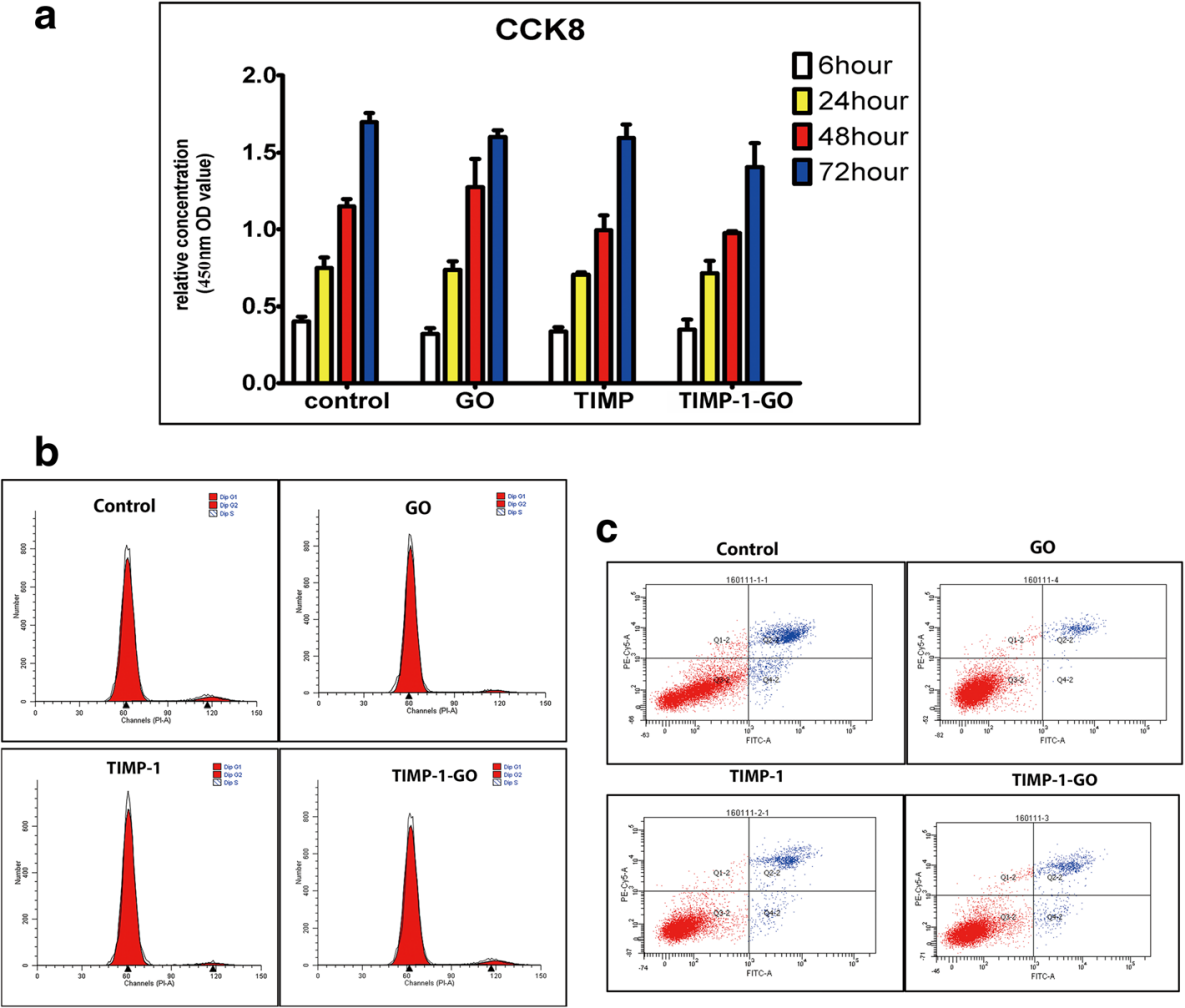
The effect of TIMP-1-GO on rat fibroblast cell proliferation and viability. **a** The viability of fibroblasts cultured in different groups show no significant differences in different time points (*p* > 0.05). **b** The cell cycle of fibroblast was not significantly different than that of cells grown in different groups (*p* > 0.05). **c** The cell apoptosis of fibroblast was not significantly different than that of cells grown in different groups (*p* > 0.05)

The proliferation and viability of rat fibroblasts cultured in control, GO and TIMP-1, were not appreciably different than those of the cells grown in the different samples of TIMP-1-GO (*p* > 0.05). The cell cycle and apoptosis of fibroblasts cultured in control, GO and TIMP-1, were not appreciably different than that of cells grown in the samples of TIMP-1-GO (*p* > 0.05) (Fig. 3a–c).

### Efficacy of TIMP-1-GO in Excisional Skin Wound Model

**Fig. 4:**
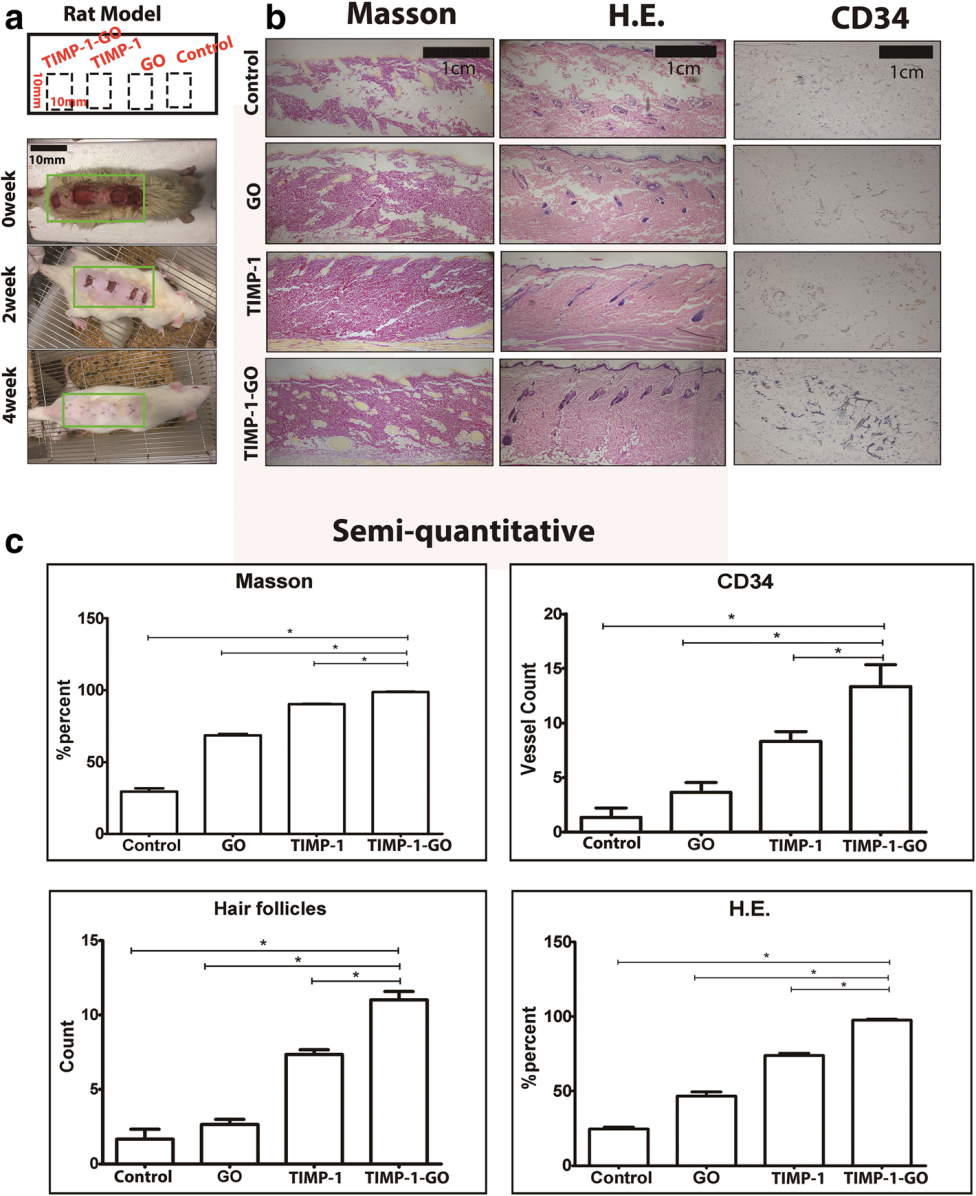
In vivo experiment. **a** Scheme and the SDS and model. **b** Histologic and immunohistochemical analysis in vivo (1 cm). Continuous collagen fiber is visible in the TIMP-1-GO group. **c** Quantitative assessment revealed significant differences between control and GO compared with TIMP-1 and TIMP-1-GO (*p* < 0.05). The hair follicles of different groups were significantly different (*p* < 0.05). Skin defects treated with TIMP-1 showed significant differences with control group and GO group by semi-quantitative (*p* < 0.05)

TIMP-1-GO was administered subcutaneously to rats to determine whether it could promote healing of the experimental wound. Four weeks after surgery, in comparison with control group, skin defects treated with TIMP-1-GO showed significant differences in terminal point (*p* < 0.05), while skin defects treated with TIMP-1 showed significant differences with the control group and GO group in terminal point (*p* < 0.05). Furthermore, the TIMP-1-GO group exhibited an enhanced therapeutic effect in hair follicles regeneration (*p* < 0.05). Skin defects treated with TIMP-1 showed significant differences with control group and GO group in terminal point (*p* < 0.05) (Fig. 4b).

### Histologic and Immunohistochemical Analysis

Histologic features of skin regeneration after treatment with PBS are displayed in Fig. 4b. The features in control groups after skin defect show broken collagen fiber visible in the specimens 4 weeks after treatment. In contrast, continuous collagen fiber is visible in the TIMP-1-GO groups at the same time point and shows a statistical difference compared with the control group. Immunohistochemical features of vascularization after treatment of TIMP-1-GO are displayed in Fig. 3c. CD34+ subcutaneous cells are visible in the specimens after 4 weeks in the TIMP-1-GO-treated groups. The quantitative assessment revealed significant differences between the control groups and the TIMP-1-GO treatment group (*p* < 0.05) (Fig. 4c).

## Discussion

In the present study, we have analyzed a potential new approach for enhancing excisional wound repair by combining recombinant TIMP-1 protein with controlled release from GO. GO has showed good biocompatibility in vitro. And TIMP-1 is shown to be continuously released from the GO vehicle for up to 40 days. The combination of TIMP-1 with GO is shown to promote the vascularization and collagen regeneration in an experimental skin defect model.

A variety of biomedical materials have been evaluated as therapy vehicles for the delivery of agents in tissue regeneration. Vehicles such as collagen, silk, titanium, calcium phosphate cement, and polylactic acid-polyglycolic acid tend to produce a rapid release of biological agent that may be undesired in some therapy settings [23]. Therefore, the ability of a biomedical vector to provide a slow continuous release of biological molecules can be seen as an important feature. A requirement for this quality is a flexible used to load the drug with the help of an electric charge [24]. Graphene oxide (GO) provides an “off-start” effect that has shown utility for the slow release of various biologic agents both in vitro and in vivo [25–27]. Here, we showed that GO can be used to exert a sustained release of recombinant human TIMP-1 protein. The interaction between hydrophobic *π* domains of GO and electrostatic interaction can activate the negatively charged domains of GO and allow efficient TIMP-1 protein absorption to GO via the inner hydrophobic regions. The long release of TIMP-1 from GO is ideally suitable for the necessary revascularization in dermal wound repair.

It is suggested that carbon particles with a concentration of more than 50 mg/ml might be deposited in tissues [28]. Wang et al. suggested that this may cause inflammatory reactions due to its extremely small diameter yet a large functional surface area of GO [29]. In contrast to previous studies, GO deposition was not detected in our study. Given the lack of an obvious side effect seen here, the potential negative of GO nanoparticles cannot be supported. Further studies have suggested on graphene including biological response and safety test [30].

The research on the effect of the interaction of the graphene oxide on cells is an important issue. Graphene is a newly developed biomedical material whose properties suggest its use in many biological applications. However, the potential biology of two-dimensional carbon structure/graphene toxicology and general biological interactions are not fully understood, which will require extensive additional studies.

## Conclusion

Graphene oxide (GO) shows sustained biocompatibility when used as a vehicle for the slow delivery of recombinant TIMP-1. TIMP-1 is shown to be continuously released from GO for up to 40 days, and the combination of TIMP-1 with GO is shown to promote the revascularization and collagen regeneration in a model of skin regeneration.
